# Prognostic relevance of androgen receptor expression in renal cell carcinomas

**DOI:** 10.18632/oncotarget.20827

**Published:** 2017-09-11

**Authors:** Sebastian Foersch, Mario Schindeldecker, Martina Keith, Katrin E. Tagscherer, Aurélie Fernandez, Philipp J. Stenzel, Sascha Pahernik, Markus Hohenfellner, Peter Schirmacher, Wilfried Roth, Stephan Macher-Goeppinger

**Affiliations:** ^1^ Institute of Pathology, University Medical Center Mainz, Mainz, Germany; ^2^ Molecular Tumor Pathology, German Cancer Research Center, Heidelberg, Germany; ^3^ Institute of Pathology, University Hospital Heidelberg, Heidelberg, Germany; ^4^ Tissue Bank, University Medical Center Mainz, Mainz, Germany; ^5^ Department of Urology, University Hospital Heidelberg, Heidelberg, Germany; ^6^ Department of Urology, Nuremberg General Hospital, Paracelsus Medical University, Nuremberg, Germany

**Keywords:** renal cell carcinoma, kidney, androgen receptor, treatment, prognostic marker

## Abstract

**Background:**

Despite rapid discoveries in molecular biology of renal cell carcinoma (RCC) and advances in systemic targeted therapies, development of new diagnostic and therapeutic strategies is urgently needed. The androgen receptor (AR) has been shown to hold prognostic and predicitve value in several malignancies. Here, we studied a possible association between AR expression and prognosis in patients with RCCs.

**Results:**

Low AR expression levels were associated with occurrence of distant metastasis and higher tumor stage in papillary and clear-cell RCCs. Importantly, multivariate Cox regression analyses revealed that AR is an independent prognostic factor for cancer-specific survival.

**Materials and Methods:**

The expression of AR was measured by immunohistochemistry and assessed by digital image analysis using a tissue microarray containing tumor tissue of a large and well-documented series of RCC patients with long-term follow-up information. Chi-squared tests, Kaplan-Meier curves and Cox regression models were used to investigate the possible relationship between AR expression and clinico-pathological characteristics and patient survival.

**Conclusions:**

Patients affected by AR-positive tumors exhibit a favorable prognosis by multiple Cox regression, while loss of AR expression is related to aggressive disease. Therefore, assessing AR expression offers valuable prognostic information that could improve treatment selection for metastatic disease. Moreover, our findings highlight a potential therapeutic use of AR pharmaceuticals in patients with RCCs.

## INTRODUCTION

Recent insights into the biology of renal cell carcinoma (RCC) have led to the introduction of novel targeted therapies for kidney cancer. Despite marked clinical effects some patients are inherently resistant and most patients eventually acquire resistance. Hence, the American Cancer Society estimates 14,240 deaths related to kidney and renal pelvic cancer in the United States for 2016 [1]. Integration of molecular information into clinical practice is certainly needed to improve and specify patient care. However, at present no biomarkers are in routine clinical use [2].

The steroid and nuclear receptor superfamily (NR) functions as DNA-binding transcription factors that regulate gene expression. Androgen receptor (AR) as member of the NR is bound to heat shock proteins which act as inhibitors, and is released and activated upon androgen binding. Ligand binding leads to AR translocation from the cytosol to the nucleus and transcriptional regulation of target genes [3]. However, also ligand independent AR activation via growth factors or cytokines has been described [4]. AR signaling interferes with the normal development and function of the target tissues, and may induce pathological conditions, including cancers [5]. Expression of the AR is detected in many tissues, mainly in male sexual organs and is essential for the development and differentiation during embryogenesis. Furthermore, AR expression is observed in the liver, cardiac muscle, uterus, urinary bladder, gastrointestinal tract, breast, and kidney [6]. However, the physiological function of the AR in these tissues still needs to be elucidated. Development and maintenance of the prostate requires androgens and AR dysregulation plays an important role in the development of prostate cancer [7]. Barboro et al. reported that a high percentage of AR-positive cells is associated with a good prognosis in prostate cancer patients [8], however there are conflicting results and the prognostic value of AR expression in prostate cancer and its clinical relevance is still debated [9]. Besides prostate cancer, AR expression has been described in a wide range of solid tumors including sarcomas, melanomas and carcinomas [10]. High AR expression is associated with lower recurrence rates and better prognosis in bladder cancer [11] and improved survival in serous carcinoma of the ovary [12], advanced squamous cell carcinoma of the head and neck [13], and breast cancer [14].

Moreover AR expression has been recognized in kidneys and kidney cancer [6, 15]. Ha et al. reported that elevated mRNA levels of AR are associated with poor prognosis in patients with localized RCC [16], whereas Zhu et al. reported that high AR expression was associated with known favorable prognostic factors, such as low pT stage and low histologic Fuhrman’s grade [17]. Similar results were described by Langner et al., who could furthermore report a favorable outcome in patients with high AR expression [18].

In this study, we systematically examined the expression of androgen receptor (AR) in RCCs and took advantage of a large hospital-based series of renal cell carcinomas with long-term follow-up information.

## RESULTS

### Immunohistochemistry

**Figure 1:**
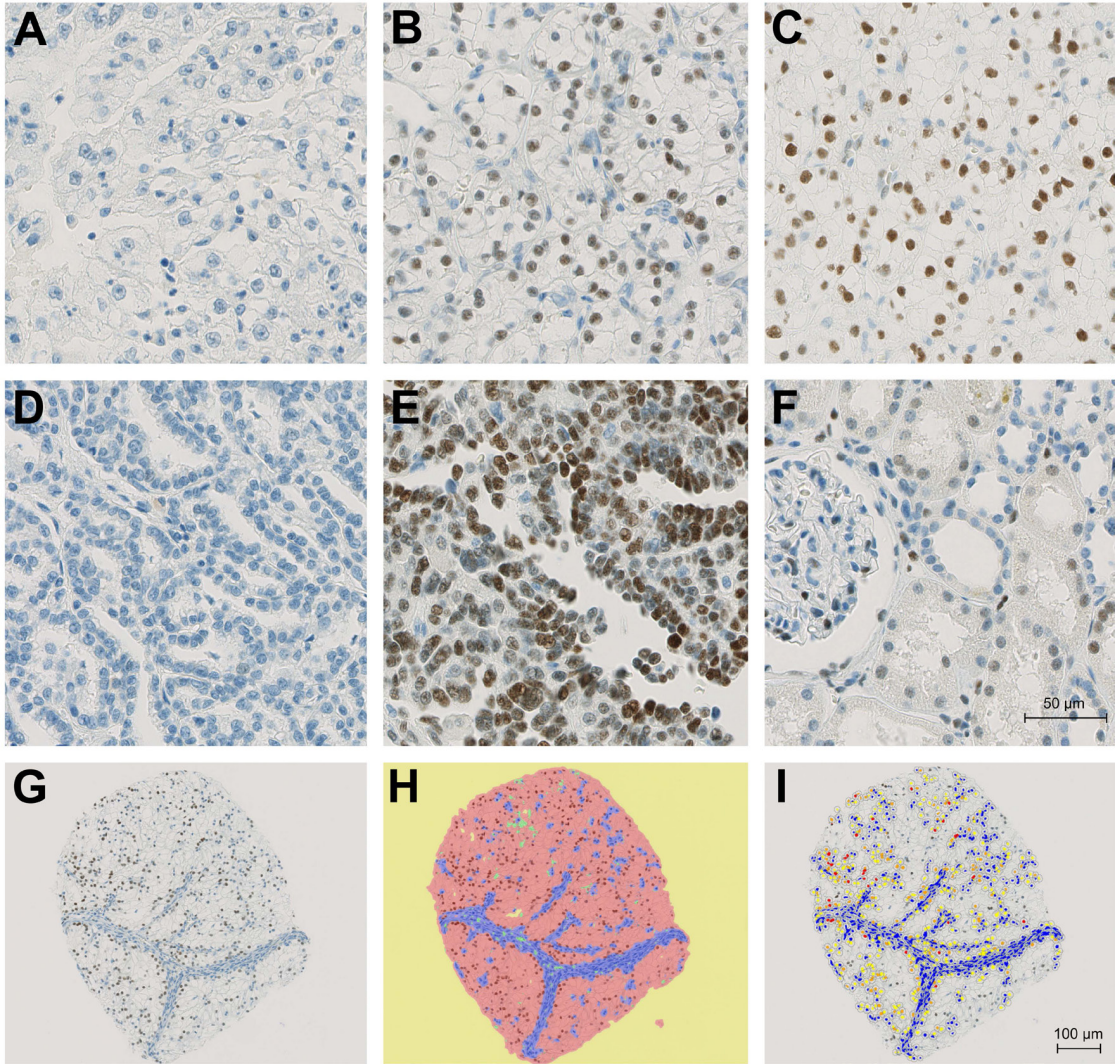
Immunohistochemical demonstration of AR expression (**A**) AR-negative ccRCC. (**B**) Moderate positivity in an subset of ccRCC cells. (**C**) Strong positivity in most ccRCC cells. (**D**) AR-negative papRCC. (**E**) Strong positivity in most papRCC cells. (**F**) Weak to moderate positivity in parietal podocytes, proximal and distal tubuli. (**G**) Representative core after IHC staining. (**H**) Core with representative classifier markup: yellow = background, red = tumor, blue = stroma, green = vessels. (**I**) Core with positive cell count markup. Blue nuclei are negative; yellow, orange and red nuclei are positive.

Immunostains were performed on tissue microarrays containing tumor and corresponding normal renal tissue from 932 patients with renal cell carcinomas. In total 546 cases could be successfully scored for AR expression by immunohistochemistry. The remaining cases were excluded from further analyses either because of insufficient tumor tissue, poor tissue preservation or missing patient information. Figure 1A–1E) depicts immunohistochemical AR expression in tumor cells. Besides, AR was also expressed in parietal podocytes, endothelial cells and variably in proximal and distal tubuli (Figure 1F).

### Clinical characteristics of the patients

The median time of follow-up was 7.67 years (mean 8.14 years, max 21.95 years). By the end of follow up 182 patients had died from RCC, median time of follow-up among these patients was 7.81 years (mean 8.14 years, max 21.88 years). The clinical and pathological features of the study population are summarized in Table 1.

**Table 1:** Clinicopathological characteristics of the study population

Variable	*n* (%)
**Study Population**	546
**Fuhrman Grade**	
1	143 (26)
2	320 (59)
3	83 (15)
**Tumor extent**	
1	307 (56)
2	41 (8)
3	177 (32)
4	21 (4)
**Local lymphnode metastasis**	
yes	38 (7)
no	508 (93)
**Distant metastasis**	
yes	89 (16)
no	457 (84)
Histologic subtype	
clear-cell RCC	477 (87)
papillary RCC	69 (13)
**Sex**	
female	208 (38)
male	338 (62)
**Age at surgery**	
> 65	232 (42)
≤ 65	314 (58)
**ECOG**	
0	332 (61)
≥ 1	214 (39)

### Digital image analysis

An average of 1595 cells could be evaluated per core. Figure 1G–1I show the analysis workflow. The results are presented separately for papillary (*n* = 69) and clear-cell (*n* = 477) RCCs.

**Figure 2:**
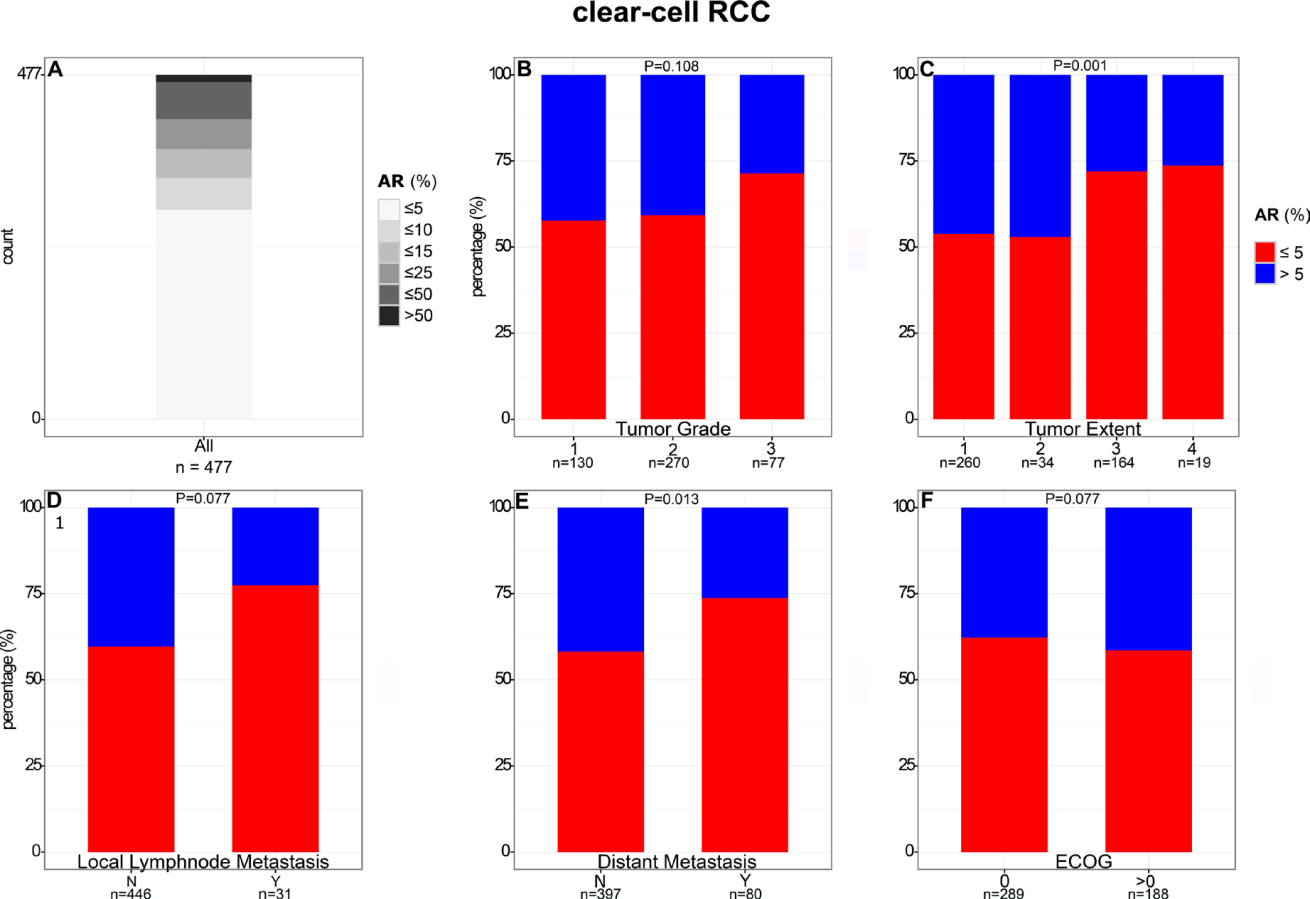
Comparison of AR expression in clear cell RCC with clinical and pathological features

The mean of positive ccRCC tumor cells was 9% (median: 3%). Maximum percentage of positive cells was 72%, minimum 0%. The majority of ccRCCs (61%, 290/477) showed no AR expression or only in a small subpopulation of tumor cells (0 to 5%). In contrast, only 10 tumors (2%) showed more than 50% AR-positive tumor cells (Figure 2).

**Figure 3:**
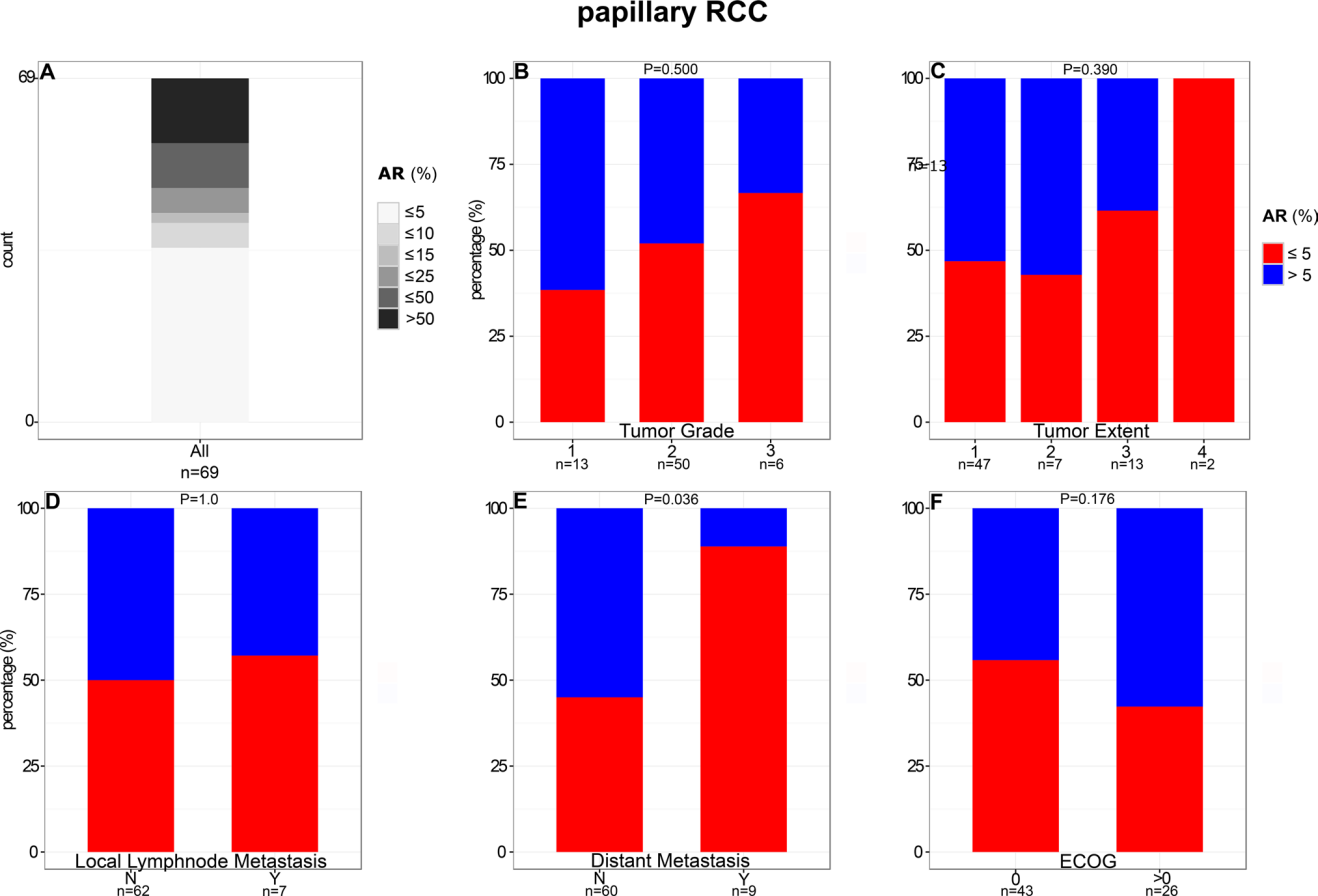
Comparison of AR expression in papillary RCC with clinical and pathological features

In contrast to ccRCC, 50% AR-positive tumor cells were detected significantly more frequently (*P* < 0.001) in papRCCs (19%; 13/69; Figure 3). The average of positive tumor cells was 23% (median: 4.78%). Maximum percentage of positive cells was 93%, minimum 0%.

### Comparison of AR expression with clinical and pathological features

The proportion of ccRCCs positive (defined as more than 5% AR-positive cells) for AR by immunohistochemistry decreased with higher tumor stage (*P* = 0.001) and presence of distant metastasis (*P* = 0.013). For example, percentage of AR-positive tumors was 46% in pT1 compared to 28% in pT3 carcinomas. No consistent association of AR expression with differentiation, lymph node metastasis, and ECOG performance status was observed (Figure 2 and Supplementary Table 2).

The proportion of AR-positive papRCCs decreased significantly (*P* = 0.036) in metastatic disease (11%) compared to localized disease (55%). Moreover, percentage of papRCCs positive for AR decreased with higher tumor stage and dedifferentiation. For instance, AR-positive tumors constitute only 33% of high-grade carcinomas compared to 61% of low-grade carcinomas (Figure 3 and Supplementary Table 3). However, this trend was not statistically significant, most likely due to limited case numbers.

Of note, there was a great intra- and interpatient heterogeneity of AR expression in normal renal tissue and high AR expression at the site of the tumor was not consistantly associated with high AR expression in the surounding kidney. If not indicated otherwise, surounding kidney was taken within < 10 mm of the tumor margin.

### AR expression and patient prognosis

**Figure 4:**
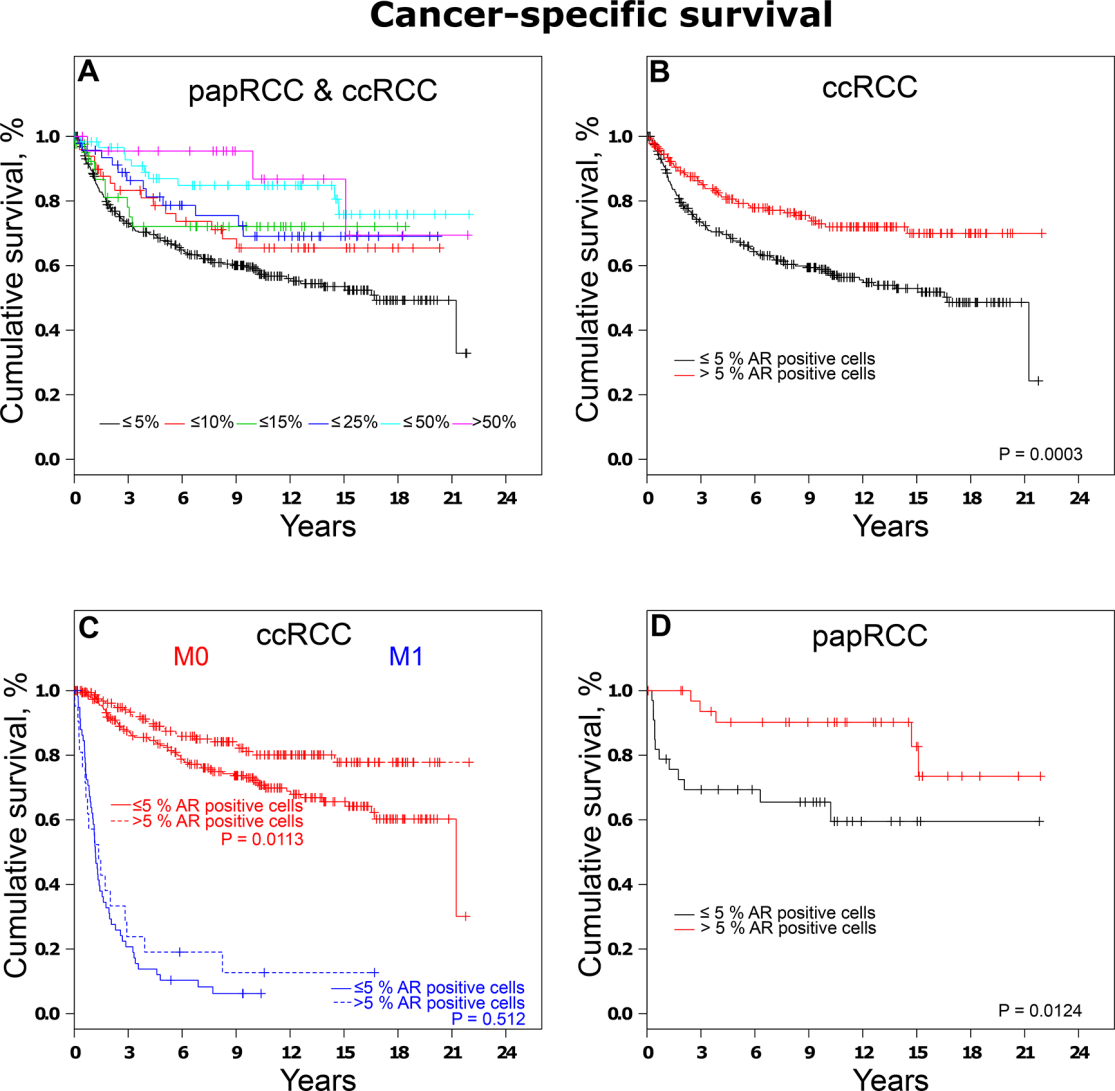
Analysis of cancer-specific survival in RCCs (**A**) Association between survival times and AR expression represented by Kaplan-Meier-Plots. (**B**) Analysis of cancer-specific survival in ccRCCs and (**C**) subset analysis of localized and distant ccRCCs. (**D**) Analysis of cancer-specific survival in papRCCs.

When tumors were grouped according to AR expression, loss of AR expression was related to shorter patient survival as depicted by Kaplan-Meier plots in Figure 4A. Subset analyses of ccRCCs and papRCCs are described subsequently.

When ccRCCs were categorized based on AR expression, univariate survival analysis revealed an increase in cancer-specific survival (CSS HR, 0.54; 95% CI, 0.38–0.76) in patients affected by tumors positive for AR compared to tumors with low/no AR expression. Subgroup analyses of non-metastatic disease also revealed a significantly higher survival rate in patients affected by AR-positive RCCs (Figure 4C).

To further validate the findings, a multivariate analysis was performed using the Cox proportional hazards model. Differences in cancer-specific survival remained statistically significant after adjustment for established prognostic factors (grade of malignancy, tumor extent, lymph node metastasis, distant metastasis, ECOG performance status, and gender) in the multiple regression analysis for ccRCC patients, and confirmed AR expression in over 5% of the tumor cells (HR 0.65; 95% CI, 0.46–0.92) as an independent favorable prognostic marker. This also applies for the subset of cases with non-metastasized ccRCCs (Table 2).

**Table 2:** Uni- and multivariate analyses of prognostic factors influencing cancer-specific survival (CSS) in clear-cell RCC

	Univariate		Multivariate					
	M0 + M1				M0		M1	
	HR (95% CI)	*P*	HR (95% CI)	*P*	HR (95% CI)	*P*	HR (95% CI)	*P*
Grade of malignancy^1^	4.46 (3.20–6.21)	**< 0.001**	1.88 (1.27–2.78)	**0.002**	**2.17** (1.28–3.71)	**0.004**	1.62 (0.94–2.77)	0.0798
Tumor extent^2^	3.63 (2.64–4.98)	**< 0.001**	2.12 (1.49- 3.03)	**< 0.001**	**2.59** (1.65–4.07)	< **0.001**	1.28 (0.72–2.26)	0.3956
Lymphnode metastasis^3^	5.06 (3.33–7.69)	**< 0.001**	1.39 (0.87–2.23)	**0.169**	**2.57** (1.32–4.99)	**0.005**	1.08 (0.57–2.04)	0.8206
Distant metastasis^4^	11.58 (8.34–16.08)	**< 0.001**	6.68 (4.62–9.67)	**< 0.001**	-	**-**	-	-
ECOG^5^	2.15 (1.58–2.93)	**< 0.001**	1.56 (1.13–2.16)	**0.007**	**1.67** (1.09–2.57)	**0.018**	1.20 (0.72–1.99)	0.4798
Sex^6^	0.69 (0.50–0.95)	**0.025**	0.76 (0.55–1.06)	**0.103**	**0.76** (0.50–1.17)	**0.219**	0.80 (0.48–1.35)	0.4034
AR-Expression^7^	0.54 (0.38–0.76)	**< 0.001**	0.65 (0.46–0.92)	**0.017**	**0.62** (0.39–0.98)	**0.040**	0.84 (0.49–1.44)	0.5180

^1^G3 vs G1/G2.

^2^pT3/pT4 vs pT1/pT2.

^3^pN1/pN2 vs N0/pN0.

^4^M1 vs M0.

^5^0 vs ≥ 1.

^6^Female vs Male.

^7^> 5 vs ≤ 5 (%).

HR, hazard ratio; CI, confidence interval. Probability values and hazard ratios considered statistically significant are shown in bold.

Similar results were observed when papRCCs were grouped based on AR expression. Hazard ratios for cases with more than 5% AR-positive cells were 0.28 (95% CI, 0.10–0.81) for cancer-specific survival. Kaplan-Meier plots are depicted in Figure 4D.

After adjustment for prognostic factors, AR expression also remained statistically significant for patients affected with papRCCs (HR 0.083 ; 95% CI, 0.02–0.43). Apart from AR expression only metastatic disease was confirmed as a significant prognostic factor, whereas grade of malignancy, tumor extent, regional lymph node metastasis, the ECOG Performance Status, gender, and age were not correlated with the clinical outcome (Table 3).

**Table 3:** Uni- and multivariate analyses of prognostic factors influencing cancer-specific survival (CSS) in papillary RCC

	Univariate		Multivariate	
	M0+M1			
	HR (95% CI)	*P*	HR (95% CI)	*P*
Grade of malignancy^1^	**2.43** (3.51–36.64)	**< 0.001**	4.71 (0.63–35.37)	0.13176
Tumor extent^2^	**12.27** (4.34–34.66)	**< 0.001**	3.18 (0.55–18.22)	0.19445
Lymphnode metastasis^3^	**20.09** (6.34–63.64)	**< 0.001**	5.99 (0.76–46.86)	0.08827
Distant metastasis^4^	**66.28** (16.28–269.8)	**< 0.001**	**14.70** (1.84–117.10)	**0.011**
ECOG^5^	1.19 (0.43–3.28)	0.732	1.71 (0.43–6.86)	0.44690
Sex^6^	0.83 (0.29–2.36)	0.724	0.38 (0.08–1.79)	0.21935
AR-Expression^7^	**0.28** (0.10–0.81)	**0.019**	0.083 (0.02–0.43)	**0.003**

^1^G3 vs G1/G2.

^2^pT3/pT4 vs pT1/pT2.

^3^pN1/pN2 vs N0/pN0.

^4^M1 vs M0.

^5^0 vs ≥ 1.

^6^Female vs Male.

^7^> 5 vs ≤ 5 (%).

HR, hazard ratio; CI, confidence interval. Probability values and hazard ratios considered statistically significant are shown in bold.

To further elucidate the relationship between AR expression and patient prognosis we took advantage of the Cancer Genome Atlas (TCGA) data on clear-cell renal cell carcinoma. Cohort used: KIRC-TCGA from 2016-01-28 [19,20]. When tumours were grouped according to AR mRNA levels (Z-score > 1.96 as up regulated; < –1.96 down regulated; between –1.96 and 1.96 not regulated) univariate survival analysis revealed that none of the patient with elevated mRNA levels (*n* = 15) died whereas 147 out of 375 patient (39.2%) without increase of AR mRNA levels deceased (*P* < 0.05), Kaplan-Meier curves are depicted in Supplementary Figure 1.

Kaplan-Meier analyses revealed, that ccRCC patients with elevated AR expression of over 5% showed a significantly longer time to progression than low/no AR expressing tumors, in papRCC-patients a trend can be observed (Supplementary Figure 2, 3).

### *In vitro* experiments

**Figure 5:**
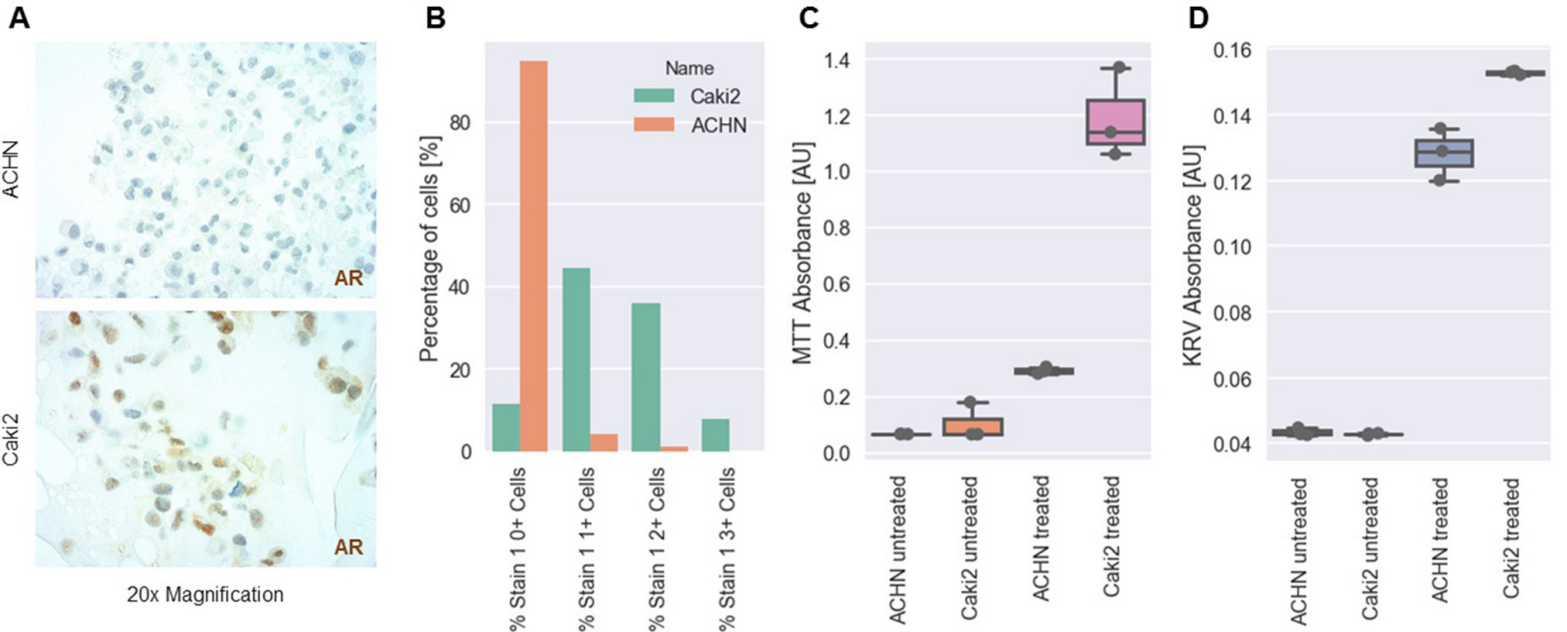
*In vitro* analysis of AR expression and treatment in RCC cell lines (**A**) Representative images of AR expression in two different RCC cell lines (ACHN: upper panel, Caki2: lower panel) as determined by IHC. (**B**) Percentage of negative (0+), weakly positive (1+), moderately positive (2+) and strongly positive (3+) cells. (**C**) MTT absorbance (cell viability) of ACHN and Caki2 cells after treatment with Cl-4AS-1, a steroidal androgen receptor agonist. (**D**) Cresylviolett (KRV) assay absorbance of ACHN and Caki2 cells after treatment with Cl-4AS-1, a steroidal androgen receptor agonist. [AU] = arbitrary unit.

To eveluate potential of AR-based treatment options, we conducted a series of *in vitro* experiments using different RCC cell lines. Using AR immunohistochemistry of cytoblocks we were able to identify Caki2 cells as AR espressing cell lines with over 80% of the tumor cells expressing the receptor. ACHN cells were found to be mostly AR negative (Figure 5A, 5B). Using Cl-4AS-1, a steroidal androgen receptor agonist, we were able to show that AR signaling led to increased cell viability and survival in MTT and Cresylviolett-assays (Figure 5C, 5D).

## DISCUSSION

Advances in genomic analyses have initiated a new era of renal cell carcinoma research, some of which have been accompanied by impressive clinical advances. Over the last decade, new targeted therapies were introduced for systemic therapy of kidney cancer and largely replaced therapies with interferon-alpha (INF-α) or interleukin-2 (IL-2). These new targeted agents show impressive initial responses, but resistance is eventually observed and cure rarely occurs [21]. Although, there are more than half a dozen novel targeted agents currently in use for metastatic disease, these therapeutics aim at only two pathways: vascular endothelial growth factor (VEGF) signaling and the mammalian target of rapamycin (mTOR). Furthermore therapy decision is still based on comorbidities, toxicity profiles, and costs [22]. Hence, there is an urgent demand for new treatment targets and biomarkers to provide additional information for patient risk stratification and molecular guided targeted therapy.

Androgen receptor is a well known therapy target in prostate cancer treatment. In fact, already in 1941 Huggins and Hodges demonstrated that hormonal manipulation can result in antitumor activity in prostate cancer [23] and medical or surgical castrating therapy is highly effective in castration-sensitive prostate cancer [24]. Furthermore androgenic compounds were also used in breast cancer therapy between the 1940s and the 1980s with sound clinical efficacy [25]. In the present study, we investigated AR expression in a large series of RCCs and compared the findings with clinical and pathological parameters. We found that AR is expressed in a substantial fraction of ccRCCs and papRCCs. Notably low AR expression was more often encountered in carcinomas with established distant metastasis. This is true for papillary and clear-cell RCCs which suggests a broader role of AR signaling in tumorigenesis of kidney cancer. Furthermore low/no AR expression was related to higher tumor extent in ccRCCs. These findings also implicate a functional relevance of AR signaling in RCC progression.

Importantly, the present study shows that patients affected by tumors with increased AR expression have a favorable clinical course with a 5-year cancer-specific survival rate of 80% in ccRCC patients (90% papRCC) compared to 67% affected by ccRCCs with low AR expression (69% papRCC). These findings are in line with previous reports based on smaller collectives, showing that AR-positive tumors are associated with a significantly better outcome [17, 18] and publicly accessible TCGA data on clear-cell renal cell carcinoma based on AR mRNA levels [19, 26]. After adjustment for other prognostic factors, multivariate analyses confirmed high AR expression as a favorable biomarker for cancer-specific survival.

Despite mounting evidence that AR participates in the tumorigenesis and/or progression of diverse tumors, the mechanisms of how AR signaling contributes to carcinogenesis are poorly understood [10]. Most prostate cancer cells express androgen receptor and androgen signaling plays a major role in the proliferation of prostate cancer cells [27]. This corresponds to a high primary response rate of hormonal ablation which is about 80% −90% [28]. However, progression of prostate cancer from the androgen-dependent to androgen-independent state secondary to hormonal therapy is a critical and a well recognized clinical problem. Some prostate cancer cells even develop an androgen-repressed phenotype and cell culture and xenograft studies indicate that androgen treatment inhibits cancer cell proliferation via Skp1, c-Myc and p27 [29–31] in this setting.

Together these studies and our data highlight differences in AR biology depending on the (tissue) context. Furthermore, variations in the methods of detection of AR expression have to be considered. We used HALO^®^ platform as a state-of-the-art, computer-based, automated digital image analysis system in order to minimize potential errors associated with manual quantification.

Our *in vitro* data demonstrate that AR signaling increased cell viability and survival in AR postive RCC cells. Interestingly, a recent study revealed that AR activation increased the proliferation rate of RCC cell lines and promoted cell migration and invasion *in vitro* and RCC progression and invasion in RCC xenografted mouse models via AR-HIFα-VEGF signaling. Importantly, ASC-J9, a newly developed AR degradation enhancer suppressed RCC progression *in vitro* and in two RCC mouse models without obvious side effects [32]. However, further functional *in vivo* and *in vitro* studies have to provide more detailed insights into the mechanisms of AR signaling in RCCs. Interestingly, a Phase II disease-oriented drug trial including 28 patients using flutamide, a synthetic, non-steroidal antiandrogen, showed partial remission in one patient and stabilization of disease in two patients [33].

In conclusion, our data demonstrate that AR is expressed in a substantial fraction of clear-cell and papillary RCCs. Correlation with clinical and pathologic findings indicate functional relevance and highlights AR expression as an independent prognostic biomarker. Further studies are needed to ascertain if AR expression is of predictive value and if new AR therapeutics are a treatment option in RCC patients.

## MATERIALS AND METHODS

### Patients

Tissue samples from 932 patients with primary renal cell carcinomas treated at the Department of Urology at the University of Heidelberg between 1987 and 2005 were collected. The human tissue samples were provided by the Tissue Bank of the National Centre for Tumour Diseases Heidelberg after approval by the Ethics Committee of the University of Heidelberg. Further details have been described previously [34].

### Tissue-micro-array

A tissue microarray containing 932 primary tumor and corresponding normal tissue samples of 932 patients was created. The tumors were graded according to the three-tiered nuclear grading system [35] and pathologically staged based on the TNM classification of 2009 [36]. Details have been described previously [37].

### Immunohistochemistry

After heat-induced antigen retrieval using the target retrieval solution ULTRA Cell Conditioning (ULTRA CC1; Ventana Medical Systems, Tucson, AZ, USA; 950–224 ) tissue microarray slides were stained with a ready to use anti-Androgen Receptor (SP107) rabbit monoclonal primary antibody (Cell Marque, Rocklin, CA, USA; 760-4605). Staining was performed using an automated staining system BenchMark ULTRA (Ventana Medical Systems) in accordance with the manufacturer’s instructions, the following solutions were used: OptiView DAB IHC Detection Kit (760–700), Hematoxylin I (790–2208), Bluing Reagent (760–2037).

### Digital image analysis

Prior to image analysis, TMA slides were digitalized using the NanoZoomer-Series Digital slide scanner (Hamamatsu Photonics, Hamamatsu, Japan). Digital image analysis was performed using the HALO^®^ platform from Indica Labs (Corrales, NM, USA) including the TMA module and the CytoNuclear v1.4 module. In short, 5–10 representative cores were used to define staining parameters such as minimum nuclear optical density (OD), minimum staining OD, nuclear and cellular size and roundness, etc. As erythrocytes showed false positivity, a tissue classifier was trained to distinguish between tumor tissue and blood filled vessels and extravasal erythrocytes. The latter were excluded from automated image analysis. The TMA module was used to automatically exclude missing or erroneous cores, further more cores with advanced tumor necrosis, scarring or non-tumor tissue were excluded by SMG manually. With the parameters fixed, the cores were analyzed and the percentage of AR-positive tumor cells was calculated. Results from automated tissue analysis were manually controlled on a set of randomly selected cores. Positive cells were defined having a minimal OD of the staining of greater than 0.090. Intensity and quantity of immunoreactive tumor cells was further calculated based on the following system: the intensity ranged from 0, negative (Min OD: 0–0.090), 1, low (Min OD: 0.090–0.190), 2, medium (Min OD 0.190–0.273), to 3, high (Min OD: > 0.273). For further analysis tumor cells were grouped in a two-tiered sytem (positive 1-3) and negative (0), to account for inter- and intraindividual variations of AR expression and to simplify the analysis. The quantity of positive tumor cells was measured continuously and was presented as positive tumor cells in relation to all cells. Further details on the analysis parameters are given in Supplementary Table 1.

### *In vitro* experiments

Cell culture of RCC cell lines (Caki2 and ACHN) were established according to standard experimental protocols. In short, Dulbecco’s Modified Eagle’s Medium (DMEM) supplemented with fetal calf serum (FCS) and antibiotics was used and cells were kept at a 40–60% confluence rate. 5 µM of Cl-4AS-1, a steroidal androgen receptor agonist (Tocris, Bristol, UK) were used to treat the RCC cell lines for 48 h with subsequent cell viability measurements. Cells with DMSO (solvant) containing medium served as controls. MTT and Cresylviolett (KRV) assays were performed according to the manufacturer’s protocols and absorbance was measured using a ELISA plate reader (Tecan Group Ltd., Maennedorf, Switzerland).

### Statistical methods

Survival was calculated from the date of nephrectomy to two different events: cancer-specific survival (CSS, event: tumor-related death, survival time was censored for patients who did not experience the investigated event) and time to progression (TTP, event: recurrence, metastasis, deaths before progression were censored).Association between survival times and AR expression was first assessed by log-rank tests and represented by Kaplan-Meier plots. In order to account for the influence of established prognostic factors, hazard ratios (HRs) and 95% confidence intervals (CIs) were adjusted for patient gender and age, tumor extent, lymph node metastasis, distant metastasis, grade of malignancy, and ECOG Performance Status in a multiple Cox proportional hazard regression. Data were analysed using the R software package (http://www.rproject.org). For count data, Fisher’s exact test (two-sided) was used. Probability values < 0.05 were considered to indicate a statistically significant result.

For mRNA data analysis voom (limma R package) was used for normalization and Z-score was calculated with formula: (value - mean normal)/SD normal.

## SUPPLEMENTARY MATERIALS FIGURES AND TABLES


