# Supplementary material for: Prognostic relevance of androgen receptor expression in renal cell carcinomas
Source: Oncotarget. 2017 Sep 11;8(45):78545–55. doi: 10.18632/oncotarget.20827 (PMC5667981; doi:10.18632/oncotarget.20827)
Supplement: Supplementary file 1 [file oncotarget-08-78545-s001.pdf]

Prognostic relevance of androgen receptor expression in renal cell carcinomas

SUPPLEMENTARY MATERIALS

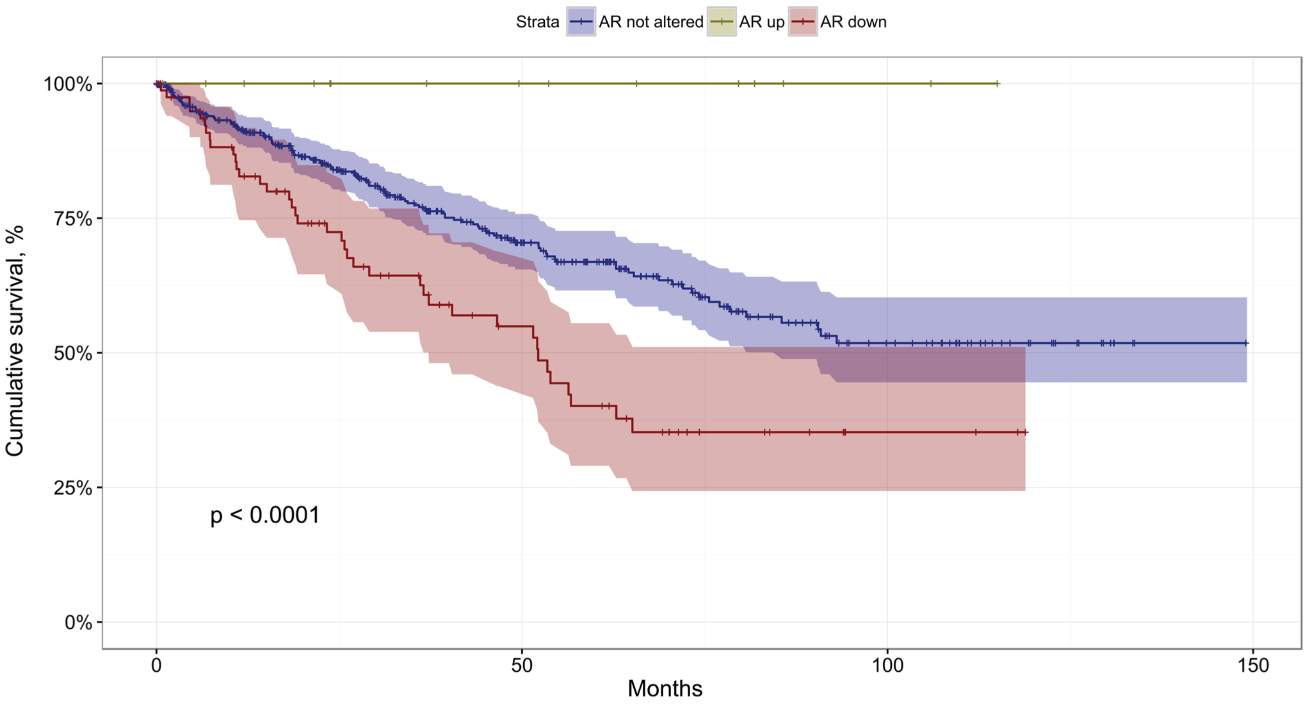

Supplementary Figure 1: Analysis of a TCGA dataset. Association between mRNA expression of AR and survival times are represented as Kaplan-Meier-Plots.

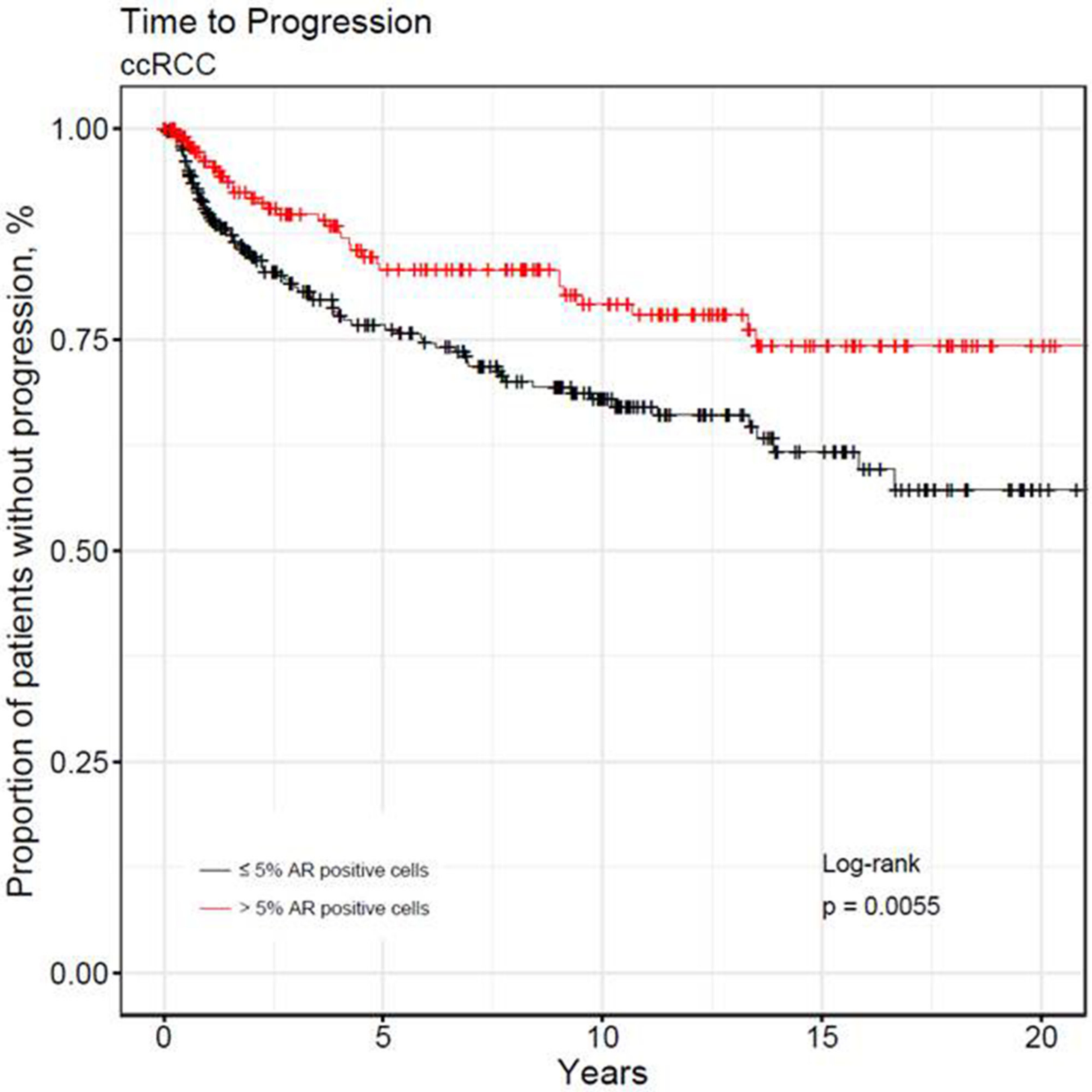

Supplementary Figure 2: Time to progression and AR expression in clear cell RCC. Association between time to progression and AR expression represented by Kaplan-Meier-Plots.

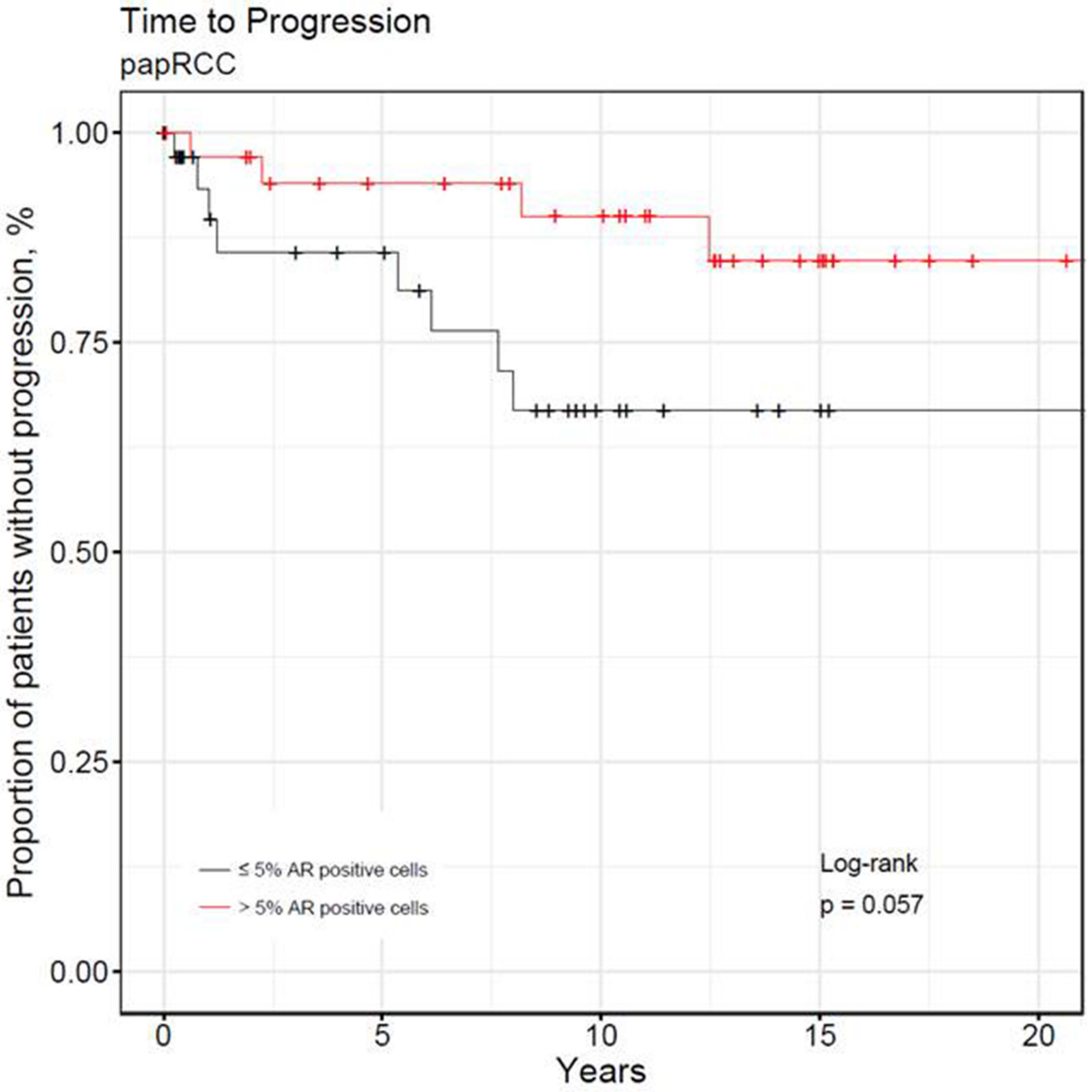

Supplementary Figure 3: Time to progression and AR expression in papillary RCC. Association between time to progression and AR expression represented by Kaplan-Meier-Plots.

Supplementary Table 1: HALO algorithm settings

Parameter Name = ”Classifier” Value = ”Foersch_AR_Ery-Elimination”
Parameter Name = ”Class List” Value = ”Tissue”
Parameter Name = ”Classify Registered” Value = ””
Parameter Name = ”Nuclear Stain” Value = ”0.251,0.184,0.130”
Parameter Name = ”Positive Stain 1” Value = ”0.310,0.438,0.587”
Parameter Name = ”Positive Stain 2” Value = ”0.0,0.0,0.0”
Parameter Name = ”Stain 1 Localization” Value = ”Nucleus”
Parameter Name = ”Stain 2 Localization” Value = ”Cytoplasm”
Parameter Name = ”Minimum Tissue OD” Value = ”3.7e-002”
Parameter Name = ”Tissue Edge Thickness” Value = ”0.”
Parameter Name = ”Nuclear Contrast Threshold” Value = ”0.466”
Parameter Name = ”Minimum Nuclear OD” Value = ”0.123”
Parameter Name = ”Minimum Nuclear Size” Value = ”9.8”
Parameter Name = ”Maximum Nuclear Size” Value = ”571.7”
Parameter Name = ”Minimum Nuclear Roundness” Value = ”0.”
Parameter Name = ”Nuclear Segmentation Aggressiveness” Value = ”0.88”
Parameter Name = ”Fill Nuclear Holes” Value = ”1”
Parameter Name = ”Maximum Cytoplasm Radius” Value = ”5.”
Parameter Name = ”Stain 1 Min OD Weak” Value = ”9.e-002”
Parameter Name = ”Stain 1 Min OD Moderate” Value = ”0.19”
Parameter Name = ”Stain 1 Min OD Strong” Value = ”0.273”
Parameter Name = ”Stain 2 Min OD Weak” Value = ”0.112”
Parameter Name = ”Stain 2 Min OD Moderate” Value = ”0.287”
Parameter Name = ”Stain 2 Min OD Strong” Value = ”0.445”
Parameter Name = ”Stain 1 Mask” Value = ”not set”
Parameter Name = ”Stain 2 Mask” Value = ”not set”
Parameter Name = ”Output Image” Value = ”Stain 1 Markup”
Parameter Name = ”Verbose Output” Value = ”Database”

Supplementary Table 2: Correlation of AR expression in renal clear cell carcinomas with clinicopathological characteristics

Variable	AR > 5%, *n*(%)	AR < 5%, *n*(%)	*P*
**Sex**			**= 0.023**
Female	61 (33)	126 (43)	
Male	126 (67)	164 (57)	
**Age at surgery, years**			= 0.138
< 65	118 (63)	155 (53)	
> 65	69 (37)	135 (47)	
**ECOG**			= 0.077
= 0	109 (58)	180 (62)	
> 0	78 (42)	110 (38)	
**Tumour extent**			**= 0.001**
Stage 1/2	136 (73)	158 (54)	
Stage 3/4	51 (27)	132 (46)	
**Fuhrman grade**			= 0.108
G1/2	165 (88)	235 (81)	
G3/4	22 (12)	55 (19)	
**Distant metastasis**			**= 0.013**
No	166 (89)	231 (80)	
Yes	21 (11)	59 (20)	
**Lymphnode metastasis**			= 0.077
No	180 (96)	266 (92)	
Yes	7 (4)	24 (8)	

Supplementary Table 3: Correlation of AR expression in renal papillary cell carcinomas with clinicopathological characteristics

Variable	AR > 5%, *n*(%)	AR < 5%, *n*(%)	***P***
**Sex**			= 0.136
Female	7 (21)	14 (40)	
Male	27 (79)	21 (60)	
**Age at surgery, years**			= 0.405
< 65	16 (47)	25 (71)	
> 65	18 (53)	10 (29)	
**ECOG**			= 0.176
= 0	19 (56)	24 (69)	
> 0	15 (44)	11 (31)	
**Tumour extent**			= 0.390
Stage 1/2	29 (85)	25 (71)	
Stage 3/4	5 (15)	10 (29)	
**Fuhrman grade**			= 0.500
G1/2	32 (94)	31 (89)	
G3/4	2 (6)	4 (11)	
**Distant metastasis**			**= 0.036**
No	33 (97)	27 (77)	
Yes	1 (3)	8 (23)	
**Lymphnode metastasis**			= 1
No	31 (91)	31 (89)	
Yes	3 (9)	4 (11)	
